# Preference of *Pentalonia nigronervosa* for infected banana plants tends to reverse after *Banana bunchy top virus* acquisition

**DOI:** 10.1038/s41598-024-53205-x

**Published:** 2024-02-05

**Authors:** Ignace Safari Murhububa, Claude Bragard, Kévin Tougeron, Thierry Hance

**Affiliations:** 1 Biodiversity Research Centre, Earth and Life Institute, UCLouvain, 1348 Louvain-la-Neuve, Belgium; 2 Applied Microbiology, Earth and Life Institute, UCLouvain, 1348 Louvain-la-Neuve, Belgium; 3 Département de l’Environnement et Sciences Agronomiques, Faculté des Sciences, Université Officielle de Bukavu, Bukavu, Democratic Republic of the Congo; 4 Faculté des Sciences Agronomiques, Université Catholique de Bukavu, Bukavu, Democratic Republic of the Congo; 5 Ecology of Interactions and Global Change, Research Institute in Biosciences, Université de Mons, 7000 Mons, Belgium; 6 Institut Supérieur d’Etudes Agronomiques et Vétérinaires de Walungu, Walungu, Democratic Republic of the Congo

**Keywords:** Ecology, Plant sciences, Zoology

## Abstract

*Pentalonia nigronervosa* Coquerel (*Hemiptera*: *Aphididae*) is the vector of the *Banana Bunchy Top Virus* (BBTV), the most serious viral disease of banana (*Musa* spp.) in the world. Before acquiring the virus, the vector is more attracted to infected banana plants in response to the increased emissions of volatile organic compounds (VOCs). Here, we test the hypothesis that BBTV acquisition directly modifies the preference of *P. nigronervosa* for infected banana plants, and that the change in preference results from the alteration of the organs linked to the VOC detection or to the behaviour of the vector. We found that the preference of *P. nigronervosa* for infected banana plants reverses after virus acquisition in dessert banana, while it remains similar between healthy and infected banana plants before and after the acquisition of BBTV. At the same time, aphids reared on infected bananas had smaller forewing areas and hind tibia length than aphids reared on healthy bananas, although the number of secondary rhinaria on the antennae was lower on dessert banana-reared aphids than plantain-reared aphids, this was not affected by the infection status of the aphid. These results support the "vector manipulation hypothesis—VMH" of pathogens to promote their spread. They have implications for the BBTV management.

## Introduction

The banana aphid, *Pentalonia nigronervosa* Coquerel (*Hemiptera*, *Aphididae*) is the vector of the *Banana bunchy top virus* (BBTV), a virus causing banana bunchy top disease (BBTD), which is the most serious viral disease of bananas worldwide^1–3^. Being a member of the family *Nanoviridae* and the genus *Babuvirus*, BBTV has a genome consisting of several segments of circular single-stranded DNA encapsulated in small isometric particles^4–7^. The disease is manifested by a general dwarfing of the plant, narrow leaves, chlorosis of the leaf margins, and dark green discontinuous streaks on the leaves, petioles and pseudotrunks. Leaves of infected plants become progressively smaller and erect, giving the plant a bushy appearance^8^.

In the plant, BBTV is restricted to phloem tissues. In infected plants, the cells surrounding the phloem vessels contain an abnormal number of chloroplasts, giving rise to the macroscopic symptoms of dark green streaks. After infection, BBTV replicates and gradually accumulates in all parts of the plant, except in leaves formed before infection, in which the virus is present, but does not replicate. This explains the fact that the vector is not able to acquire the virus from these leaves^9,10^. BBTV is transmitted after a sap uptake by the aphid vector on an infected plant, in a persistent, circulative and non-propagative manner^10,11^. Being a persistent virus, acquisition of BBTV requires prolonged feeding (hours) on an infected plant. Virions pass from the insect gut into the hemolymph and eventually salivary tissues (hence “circulatory transmission”), without replicating in the vector (hence “non-propagative”)^12,13^.

In certain aphid species, the search for and selection of the host plant are mainly facilitated by the volatile organic compounds (VOCs) emitted by the plants^14–17^. The profiles of these VOCs can vary in quality and quantity with the infection by phytoviruses. For example, Safari Murhububa et al.^18^ showed that *P. nigronervosa* was more attracted to infected banana plants than to healthy ones, through an increased in VOC emission. The preference of vectors to infected plants, as is the case for BBTV, could contribute to increased virus spread. However, a preference for infected plants will accelerate the spread of BBTV only when infected plants are rare, not when they become widespread in a plant population^19,20^. In this case, to facilitate the spread of the BBTV, it would be necessary that, once the virus acquired, the vector will be deterred from infected plants and attracted to healthy banana plants.

Indeed, the interaction between the plant and the pathogen often produces a feedback effect on the vectors. While non-infectious vectors sometimes prefer infected plants, infectious vectors tend to prefer uninfected hosts favouring transmission and global spread^20–24^. This is the case in the study by Rajabaskar et al.^21^, where non-virulent *My. persicae* preferred to settle on *Barley yellow dwarf virus* (BYDV)-infected potato plants compared to uninfected plants, while viruliferous *My. persicae* (carrying *Potato leafroll virus*-PLRV) preferentially settled on uninfected potato plants compared to infected plants. Similarly, in the in vitro study by Ingwell et al.^20^, the aphid *Rhopalosiphum padi*, after acquiring the *barley yellow dwarf virus* (BYDV), preferred uninfected wheat plants, while the non-infecting aphid preferred BYDV-infected plants. This change in behaviour should favour the spread of the virus since the preference of non-infectious vectors for infected plants will favour acquisition, while the preference of infectious vectors for non-infected hosts will favour transmission. Natural selection on the parasite or pathogen has favoured the ability to induce host behaviour that enhances its transmission, which is usually referred to as the vector manipulation hypothesis (VMH)^20,25^.

The question is thus how BBTV can modify the behaviour of its host regarding to plant attractivity although its no-propagative nature. As BBTV does not multiply in the aphids, we hypothesize that during development on an infected plant, BBTV may act through a change in the plant quality on the alates (winged aphids), even if non-propagative viruses also interact with the vector at the cellular level during movement between tissues and organs and may potentially influence the physiology and behaviour of the vector^26^.

In previous work, we have demonstrated that infection of banana with BBTV enhances the reproductive capabilities of *P. nigronervosa*, despite the decrease in the size of aphids reared on infected banana plants^17^. Moreover, other studies have reported that the size of an aphid can also vary according to virus infection^27^. On the other hand, aphids have olfactory receptor systems responsible for the detection of plant VOCs, through olfactory sensilla including primary and secondary rhinaria on the antennae^28,29^, as shown in several electrophysiological studies^30–34^, and a flight system allowing dispersal of winged forms to other plants^33^.

Our aims was thus to determine the consequences of BBTV acquisition, during development on an infected plant, on the selection behaviour of banana plants by *P. nigronervosa* as well as on the wing size and antennal secondary rhinaria of the alates, knowing that it is the winged morphs that are actually responsible for the transmission of phytoviruses, and to this end are equipped with an elaborate sensory system for detection, flight and localization of host plants^35^. As in Safari Murhububa^18^, healthy and infected seedlings of two of the most representative banana varieties in the world: Cavendish dessert banana (AAA genome) and Pacific plantain (AAB genome)^36–38^ were used in this work.

## Materials and methods

### Insects and plants

The *P. nigronervosa* colony was obtained from parthenogenetic females collected from a healthy banana plant in the province of South Kivu (Democratic Republic of the Congo), and then continuously reared on live banana plants, free of any disease, and planted in pots (Thermoformed red MCI 17:2L) on a potting soil substrate. The aphid-banana plant combination was maintained in cages (200 × 100 × 100 cm) of small-mesh netting, placed in air-conditioned chambers at 25 ± 2 °C, a relative humidity of 40 ± 5%, and an artificial photoperiod of 12/12 h. Alates (viruliferous and non-viruliferous) were obtained when aphid population density increased significantly, or when banana quality decreased significantly^35,39^.

As *P. nigronervosa* transmits the BBTV virus in a non-propagative manner^10,11^, all newly produced nymphs are non-viruliferous. The viruliferous winged adults used in this work were therefore reared on infected plants throughout their lives (from nymph production to winged adult) to ensure that the colonies contained only viruliferous individuals. An adult female was deposited on a live plant infected with BBTV, and was carefully removed from the infected plant 24 h later, along with the excess nymph produced, leaving only one nymph from which five clonal colonies (corresponding to one colony per plant) of viruliferous winged aphids were obtained. The colonies of non-viruliferous winged aphids used in this work were obtained in the same way from healthy plants. Five colonies of viruliferous and non-viruliferous aphids were considered in this mass aphid survey. Twenty winged aphids, including ten taken at random from an infected banana plant and ten taken at random from a healthy banana plant, were tested by PCR to confirm the acquisition of BBTV (Supplementary Figure S1). All aphids sampled from the infected plant were found to be viruliferous and all aphids sampled from the healthy plant were found to be non-viruliferous.

The plant material consisted of dessert banana plants of the cultivar Cavendish (strict triploid *M. paradisiaca*—AAA), and plantains of the cultivar Pacific (hybrids and triploids *M. balbisiana*—AAB), either symptomatic (with symptoms of BBTV) or asymptomatic (without symptoms of the disease). Four treatments were considered: 1° aphids reared on healthy dessert banana (HDB) and 2° healthy plantain banana (HPB), 3° aphid reared on infected dessert banana (IDB) and 4° infected plantain banana (IPB). The plants were identified and collected from peasant plantations in the province of South Kivu in (Democratic Republic of Congo), with the support of the International Institute of Tropical Agriculture-IITA/Kalambo (Bukavu, DR Congo), in accordance with Dowiya et al.^40^, then certified by the Plant Clinic of the 'Agro Louvain-Services' platform (located in the plant pathology laboratory of the Université catholique de Louvain, Louvain-la-Neuve, Belgium). This study therefore complies with local regulations and guidelines in the Democratic Republic of Congo.

As transmission of the virus by mechanical inoculation has never been successful^41,42^, all the plants used including the healthy ones were maintained and propagated in the tropical greenhouse (local n°13; G2) of the UCLouvain (Louvain-la-Neuve, Belgium) using the PIF technique (Plants from Stem Fragments)^43–47^ and were irrigated daily, until they reached 40–60 days of age (4–6 leaf stage), for their use in aphid rearing, as well as for viruliferous and non-viruliferous alate aphid attractiveness tests. Severe symptoms of BBTV were observed for plants obtained directly by the PIF technique from infected banana bulbs, since offspring from an infected strain are automatically infected^48,49^. Plants were tested twice by PCR to confirm the genotype (Supplementary Figure S2) and infection status (Supplementary Figure S3) of each plant.

### Choice test to assess aphid preferences

In this experiment, both viruliferous and non-viruliferous alates were used to assess the attractiveness of *P. nigronervosa* to different types of banana plants. This was achieved following methods based on Safari Murhububa et al.^18^, by using a short-range aphid flight device in a wooden cage (200 × 100 × 100 cm), the front of which was covered with a fine-mesh fabric to facilitate experimental handling. Each test was repeated 20 times, and at each repetition, two types of live banana plants (never used before) of each genotype at the 4–6 leaf stage (≈ 50 cm high) were placed in the cage. Twenty alate aphids reared from larval deposition to adult on the infected plants (viruliferous aphids) or on healthy plants (non-viruliferous aphids), were placed in an open Petri dish, on the other side of the cage and at an equal distance from the two plants (≈ 15 cm), to test their choice between the two olfactory sources. For each of the two aphid types, four tests were performed: HDB–HPB, HDB–IDB, HPB–IPB and IDB–IPB. Two further tests were carried out as controls for each of the two aphid types: HDB–Soil (pot containing soil alone), and HPB–Soil.

The aphid choices were evaluated by counting the number of aphids found on each of the two types of banana plants 24 h after their deposition in the cage. The banana plants and the viruliferous and non-viruliferous alates used in each replicate and treatment had never been used before.

### Measurement of aphids

To assess the consequences of plant quality when infected by BBTV on VOC detection by aphids, the secondary rhinaria (SR) on the third, fourth and fifth antennal segments (segments that bear SR in *P. nigronervosa*)^50^ were counted on aphids from healthy and BBTV-infected dessert and plantain banana plants. Forewing area was also measured on the same individuals. Altogether, 20 aphids (20 replicates) per treatment (totaling 80) alate aphids from four different types of banana plants (treatments) were assessed. The hind tibia length (mm) of theses alate aphids, representative of body size^51^ was measured as a co-variable to assess its respective correlations with forewing area and total SR numbers. Forewing area and hind tibia length were measured under a camera-mounted stereomicroscope (LEICA MZ6), while SR counts were made using a camera-mounted light microscope.

### Statistical analysis

Comparisons of decisions made by viruliferous and non-viruliferous alate aphids between each pair of olfactory sources were made using Student's t-tests (the normal distribution of our data was visually assessed and tested using the Shapiro–Wilk test). Differences in total SR numbers, forewing areas, and hind tibia length between each treatment were analyzed using generalized linear models (GLM) fitted with a Poisson family or a Gaussian family, respectively. We used the interaction between the genotype and the infection status as fixed terms in the models. The tibia length was used as a covariate in the models testing for differences in wing area and SR numbers. Contrasts (estimated marginal means (EMMs) between levels of a significant variable (p < 0.05) were analyzed using the emmeans package^52^. Pearson’s correlations test between tibia length and both wing area and SR number were done, using a Holm adjustment method. Statistical analyses were performed on R v4.0^53^.

## Results

### Choice test to evaluate attractiveness of non-viruliferous and viruliferous alates

**Figure 1:**
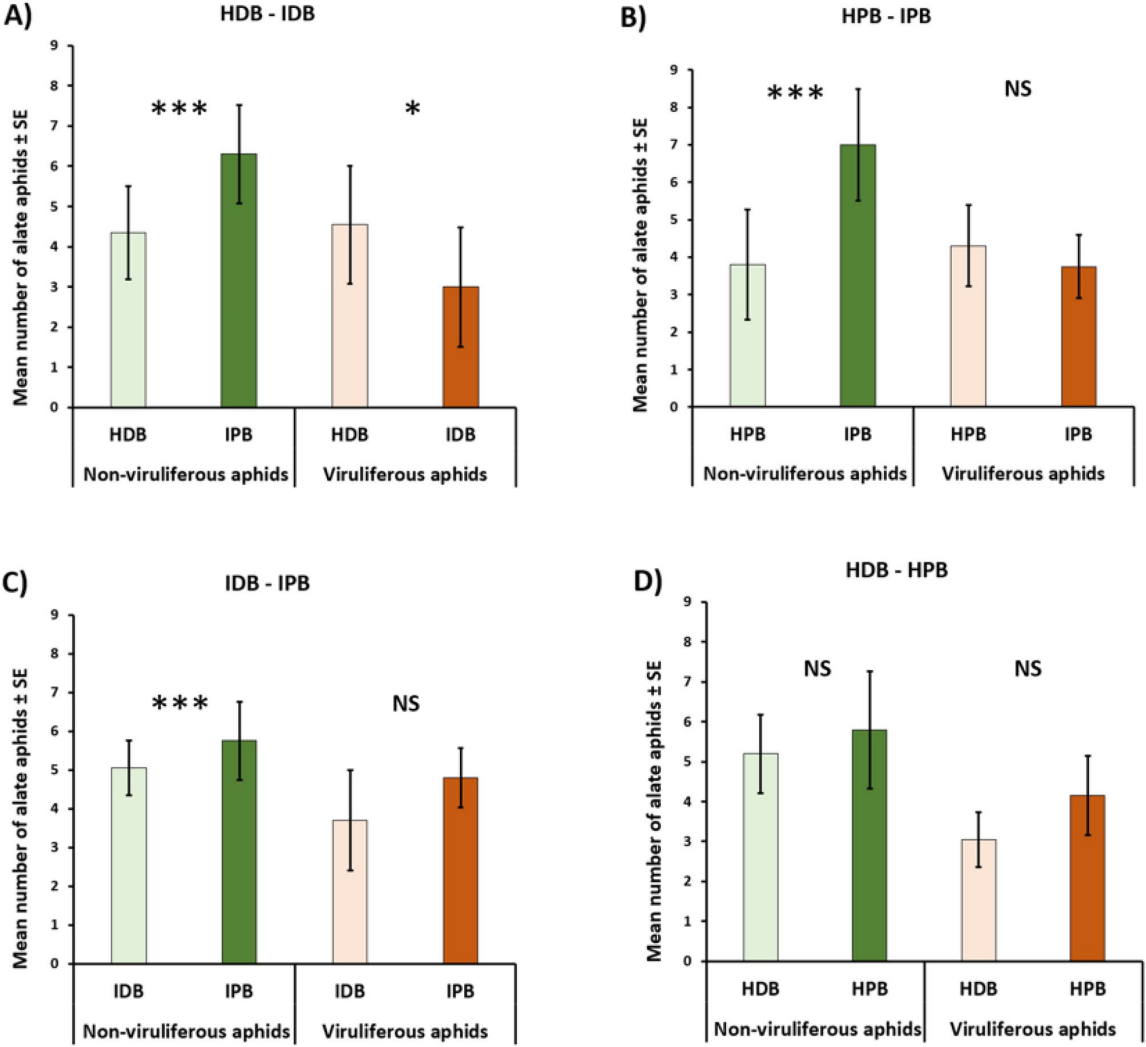
Comparison of the choice of viruliferous and non-viruliferous alate aphids on banana plants. The average number of aphids per plant is shown with standard deviation, (N = 20 replicates, each time with 20 aphids per experiment). *NS* not significant, (*): p value < 0.05, (***): p value < 0.001. (**A**) Healthy dessert banana and infected dessert banana, (**B**) healthy plantain and infected plantain, (**C**) infected dessert banana and infected plantain, (**D**) healthy dessert banana and healthy plantain. *HDB* healthy dessert banana, *IDB* infected dessert banana, *HPB* healthy plantain banana, *IPB* infected plantain banana.

In validation tests comparing the attractiveness of banana aphids towards a banana plant or a control (potting soil only), non-viruliferous and viruliferous aphids were each time more attracted to the banana plant (Supplementary Table S2). Viruliferous aphids were more attracted to HDB than to IDB (t = 3.37, p = 0.011, Fig. 1A), while non-viruliferous aphids were more attracted to IDB than to HDB (t = − 5.04, p < 0.001, Fig. 1A). Non-viruliferous were more attracted by IPB than by HPB (t = − 6.68, p < 0.001, Fig. 1B), but viruliferous aphids did not discriminate between IPB and HPB (t = 0.75, p = 0.38, Fig. 1B). Viruliferous aphids were similarly attracted by IDB and IPB (t = 2.85, p = 0.091, Fig. 1C), while non-viruliferous ones were more attracted to IPB than to IDB (t = − 2.65, p = 0.01, Fig. 1C). Neither viruliferous nor and non-viruliferous aphids discriminated between HDB and HPB (t = − 1.48, p = 0.15; t = 3.37, p = 0.66; Fig. 1D).

### Changes in morphology related to flight and VOC detection

**Figure 2:**
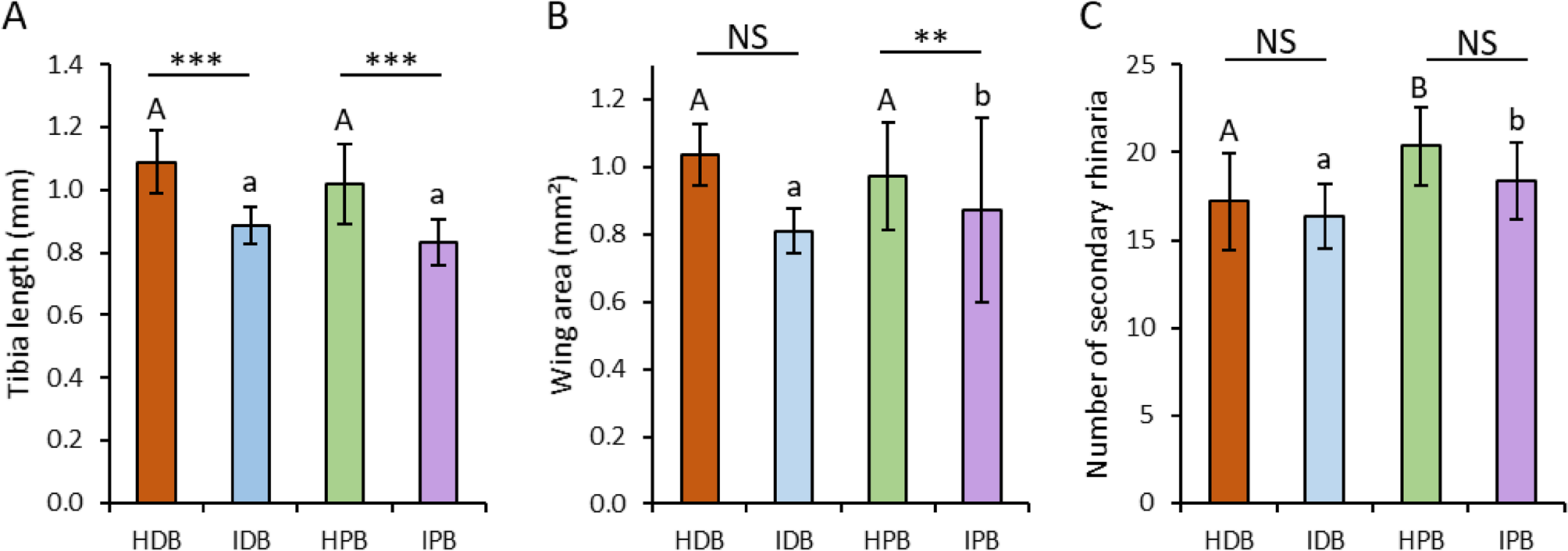
Effect of BBTV on (**A**) average length of hind tibiae (mm), (**B**) average forewing area (mm^2^) and (**C**) average number of SR on antennae of aphid reared on the four kind of banana plants: *HDB* healthy dessert banana, *IDB* infected dessert banana, *HPB* healthy plantain banana, *IPB* infected plantain banana. Means with standard errors (N = 20). Statistical results (GLM): NS indicate non-significant results (p > 0.05), stars indicate significant differences (p < 0.05) between infection statuses (for each genotype) and different letters indicate significant differences (p < 0.05) between genotypes (upper case letter for healthy plants and lower case letter for infected plants).

The length of the posterior tibiae of alates varied with virus infection, with longer hind tibiae in alate aphids on healthy banana plants (non-viruliferous aphids) than on infected plants (viruliferous aphids) (GLM: LR = 84.3, p < 0.001) for both the dessert genotype and the plantain genotype (EMMs: t-ratio = 6.82, p < 0.001 and t-ratio = 6.17, p < 0.001, respectively) (Fig. 2A), and did not vary between genotypes, for both infection status (LR = 0.21, p = 0.64) (Fig. 2A).

After controlling for the tibia size, there was a marginally significant interaction effect between the infection status and the banana genotype on forewing area (GLM: LR = 3.33, p = 0.06). For plantain genotypes, wing area was higher in aphids on healthy plants than on infected plants (t-ratio = − 2.50, p < 0.05), but that was not the case for dessert banana plants (t-ratio = − 0.37, p = 0.71). Aphids on the plantain banana variety had larger wing area than on the dessert variety when developing on infected plants (t-ratio = − 3.15, p < 0.01), but not on healthy plants (t-ratio = − 0.60, p = 0.56) (Fig. 2B).

After controlling for the tibia size, alate aphids reared on plantain bananas had a higher number of SR than aphids reared on dessert banana plants (GLM: LR = 7.78, p < 0.01), for both infection statuses (z-ratio = − 2.38, p < 0.05 and z-ratio: − 1.67, p < 0.05, for healthy and infected bananas, respectively). There was no effect of the infection status on the number of SR (GLM: LR = 0.34, p = 0.56), for both the dessert (z-ratio = 0.14, p = 0.88) and the plantain genotypes (z-ratio = 0.76, p = 0.45) (Fig. 2C).

**Figure 3:**
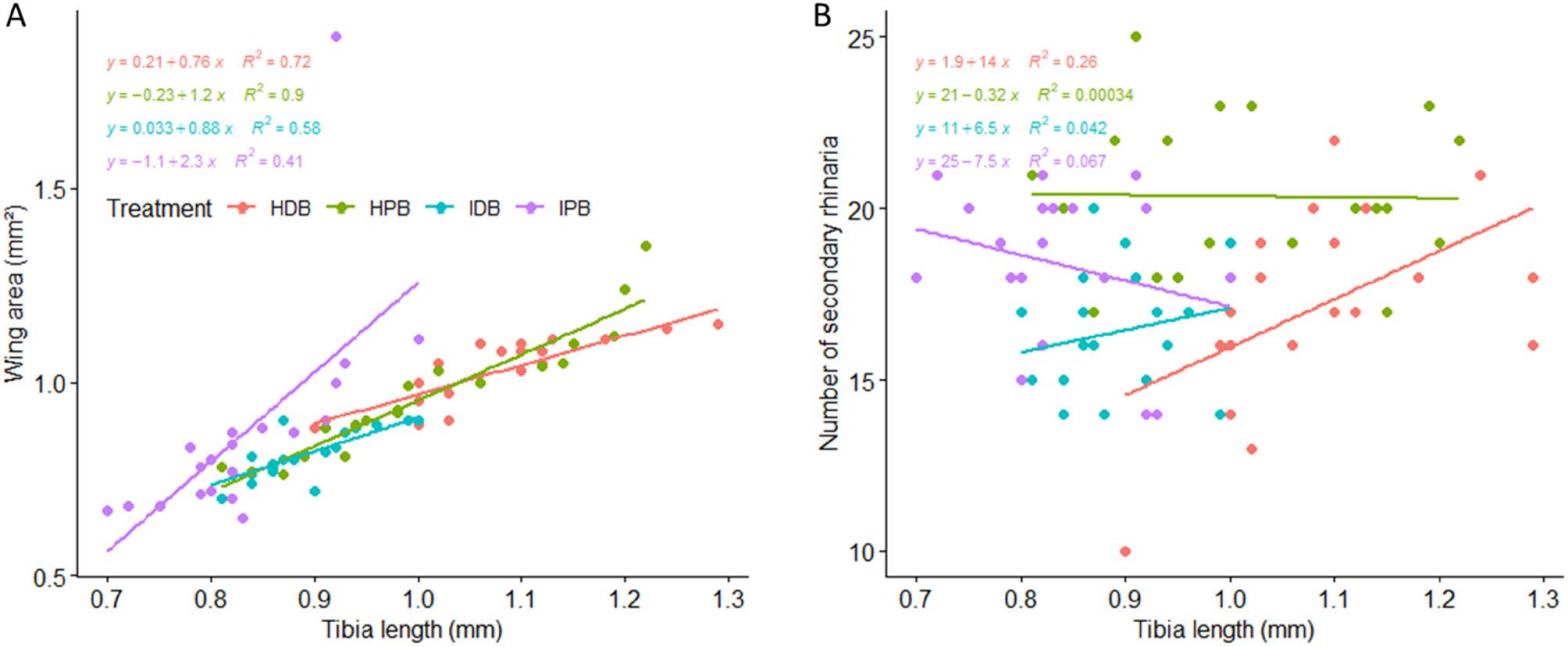
Linear correlation (n = 20) between (**A**) forewing area and hind tibia length, (**B**) total number of secondary rhinaria on antennae and hind tibia length of *P. nigronervosa* alates, collected from *HDB* healthy dessert banana, *IDB* infected dessert banana, *HPB* healthy plantain banana, *IPB* infected plantain banana. Regression line equation and R^2^ is indicated for each correlation.

There was a strong linear correlation between hind tibia size and forewing area in all four treatments; HDB (t = 6.76, p < 0.001), IDB (t = 5.58, p < 0.001), HPB (p < 0.001, t = 13), and IPB (p < 0.001, t = 7.37) (R^2^ and regression equations provided in Fig. 3A). There was also a linear correlation between the size of the hind aphid tibia and the total number of their antennae SR, but only in one of the four treatments; HDB (t = 2.54, p = 0.041), IDB (t = 0.89, p = 0.63), HPB (t = − 0.08, p = 0.99), and IPB (t = − 1.14, p = 0.27) (R^2^ and regression equations provided in Fig. 3B).

## Discussion

In the present study, we evaluated the direct effect of BBTV acquisition on the selection behaviour of banana plant by *P. nigronervosa*, and on the wing size, tibia size and number of antennal SR of alates. We found that when reared on healthy plants, *P. nigronervosa* was more attracted to infected dessert banana than to healthy dessert banana, and to infected plantain banana than to healthy plantain banana plant. When reared on an infected plant, alates became more attracted to healthy dessert banana than to infected dessert banana plants, while no significant differences were recorded for healthy and infected plantain banana plants. The preference of *P. nigronervosa* for infected banana plants therefore tends to be reversed, or at least lost in case of plantain genotype, after BBTV acquisition. In consequence, this change in attractivity may promote or at least not prevent aphid movement toward heathy plants after virus acquisition. It may thus increase the probability of virus transmission of healthy plants. In various pathosystems, it is observed that virus spread can be consistently accelerated if vector preferences are dynamic, such that vectors preferentially settle and feed on infected plants until they have acquired the virus, after what they preferentially settle and feed on uninfected plants^17^. These results are consistent with the vector manipulation hypothesis (VMH) which states that plant pathogens enhance their spread to new hosts through their effects on mobile vectors^17,54^. It has been shown, for example, that non-viruliferous *R. padi* prefer BYDV-infected wheat plants over sham-inoculated plants, but aphids preferred uninfected plants after having acquired BYDV^20^. The preference of an aphid vector of phytovirus towards their hosts is therefore dynamic, depending on whether it is in the pre- or post-acquisition period^20,24,55^.

The effects of the virus on phenotypes show a remarkable degree of convergence between viruses whose transmission is favoured by the same vector behaviour. This convergence is based more on transmission mechanism than on phylogeny^20^. These vector manipulation traits, dependent on the transmission mechanism, appear to be adaptive and not just by-products of infection^24,56–58^. For example, non-persistently transmitted viruses bind transiently to insect mouthparts, and interactions in these pathosystems are likely to be limited to indirect effects on the vectors^26^. In this strategy, the vector appears to be falsely attracted to the infected plant and then rapidly disperses^59,60^. To optimize its transmission and spread, the non-persistent virus induces a pull–push strategy from its vector to its host plant. In contrast, persistent transmission viruses attract their vectors to infected plants more frequently and improve their performance on these plants compared to non-persistent transmission viruses. In the persistent strategy, the virus vector develops a strong affinity with the host plant. Acquisition of persistently transmitted viruses requires sustained feeding in the phloem of the infected plant, virus particle circulation in the insect, and accumulation in accessory salivary glands. These viruses are almost exclusively dependent on the vector for transmission^25^. After acquisition, the insect remains a vector for life^23,61^. Although they do not replicate in the vector, persistently transmitted viruses interact with the vector at the cellular level during movement between tissues and organs, with the potential to directly alter the physiology and behaviour of the vector^26^. The effects of pathogens on vector biology and behaviour have been documented in several pathosystems^62,63^. We believe that alterations in *P. nigronervosa* behaviour that have fed on BBTV-infected plants are primarily due to virus acquisition rather than the effect of the infected plant phenotype. This was confirmed in aphids which acquired the virus without contact with infected plants but by feeding on a medium containing viral particles, thus circumventing the potential indirect effect of the plant, and attesting to a direct effect of a plant virus on the aphid vector^20^.

However, the present study does not demonstrate that the alteration of the physiology of *P. nigronervosa* is exclusively or at all due to the direct effect of BBTV on the vector, especially as BBTV does not replicate inside *P. nigronervosa*. In fact, some phytoviruses do not replicate in the vector, so that these vectors are not pathogenic hosts, sensu stricto. Our model pathogen (BBTV) is a member of the *Nanoviridae*, transmitted by *P. nigronervosa* in a persistent, circulative, and non-propagative mode for which evidence of multiplication (replication and transcription) within the vector has never been clearly observed^64^. Nevertheless, a study by Siscard et al.^65^ on another nanovirus (*Faba bean necrotic stunt virus*-FBNSV) detected changes in virus genotypes within the vector. Even that is not evidence of direct vector manipulation, so it does not help the argument here. Several studies have shown that viruses with circulatory and non-propagative transmission can directly modify the biology and behaviour of vectors. For PLRV, it has been shown that non-viruliferous aphids are attracted to infected hosts, while viruliferous aphids are attracted to healthy plants^20,21^. In Moreno-Delafuente et al.^66^ whiteflies that acquired TYLCV remained immobile longer and fed more on phloem than whiteflies that did not acquire the virus. The mechanisms behind these interactions are still largely unknown. Patton et al.^67^ contributes to the understanding of plant-vector-pathogen interactions, by elucidating mechanisms by which in a circulative–non propagative phytoviruses can manipulate host plants. This study revealed that total free amino acids increased in virus-infected leaves, and that at least three individual proteins of PLRV (P0, P1 and P7) are thought to modify plant-vector-pathogen interactions through changes in aphid induction of ethylene and jasmonic acid, by the presence of aphids. Such studies are essential for an understanding of the *BBTV-P. nigronervosa-Banana* interaction.

We also found that plant virus infection caused a decrease in the length of the hind tibiae and forewing area (but not the SR) of aphids reared on both banana genotypes. This suggests that BBTV infection has a negative impact on aphid growth, probably through plant quality. Viral infection of banana plants thus leads to a fecundity-body size trade-off in *P. nigronervosa*, in accordance with previous results of Safari Murhububa et al.^68^. Many studies have reported trade-offs between fecundity and other traits in insects^69,70^. As BBTV does not replicate in *P. nigronervosa*, alterations in vector physiology are logically due to changes in the phenotypic characteristics of banana plants induced by virus infection, rather than virus acquisition. When reared on an infected plant, the aphid allocates its energy reserves (lipids, fats and carbohydrates) to maintain its reproduction to the detriment of its own size.

Although the number of studies dealing with direct effects or direct manipulation of behaviour linked to virus infection is starting to increase, this kind work is very recent, and it is still too early to define a global view and develop a general paradigm on this subject. This work contributes to a better understanding of the plastic responses of BBTV manipulation of *P. nigronervosa*, leading to disease progression. All the same, these results may help to develop a new ecological strategy to prevent the colonization of banana plants by *P. nigronervosa*, and thus avoid transmission of BBTV to banana plants.

### Supplementary Information

Supplementary Information.

## Data Availability

Some of our datasets related to the "Choice test to assess aphid preferences" section of the current study were used in Safari Murhububa et al.^18^ in 2020 and are therefore no longer publicly available. However, the raw data relating to the "Aphid Measurement" section of the current study can be found in the supplementary material (Supplementary Table S1).

## References

[1] Thomas JE, Iskra-Caruana ML, Jones DR (2000). Bunchy top. Diseases of Banana, Abaca and Ensete.

[2] Chandrassekar A, Kalaiponnani K, Elayabalan S, Kumar KK, Angappan K, Balasubramanian P (2011). Screening of *Banana bunchy top virus* through multiplex PCR approach. Arch Phytopathol. Plant Prot..

[3] Qazi J (2016). *Banana bunchy top virus* and the bunchy top disease. J. Gen. Plant Pathol..

[4] Burns RM, Dale HJL (1995). The genome organization of *Banana bunchy top virus*: Analysis of six DNAss components. J. Gen. Virol..

[5] Timchenko T, Bernadi F (2007). Nanoviruses, small plant viruses: Similarities and differences with geminiviruses. Virology.

[6] Stainton D, Martin DP, Muhire BM, Lolohea S, Halafihi M, Lepoint P, Blomme G, Crew KS, Sharman M, Kraberger S, Dayaram A, Walters M, Collings DA, Mabvakure B, Lemey P, Harkins GW, Thomas JE, Varsani A (2015). The global distribution of *Banana bunchy top virus* reveals little evidence for frequent recent, human-mediated long distance dispersal events. Virus Evol..

[7] Mukwa LFT, Gillis A, Vanhese V, Romay G, Galzi S, Laboureau N, Kalonji-Mbuyi A, Iskra-Caruana L, Bragard C (2016). Low genetic diversity of *Banana bunchy top virus,* with a sub-regional pattern of variation, Democratic Republic of Congo. Virus Genes.

[8] Gatsinzi, F. Les Principales Maladies et Ravageurs du Bananier au sein de la CEPGL in Séminaire sur les maladies et ravageurs des principales cultures vivrières d’Afrique centrale. Bujumbura (Burundi): Iraz, CTA (1987).

[9] Hafner GJ, Harding RM, Dale JL (1995). Movement and transmission of *Banana bunchy top virus* DNA component one in banana. J. Gen. Virol..

[10] Iskra-Caruana, M. L. *Banana bunchy top virus*—BBTV, Analyse du risque Phytosanitaire (ARP), CIRAD, BAN-v1, 31 (2003).

[11] Anhalt MD, Almeida RPP (2008). Effect of temperature, vector life stage and plant access period on transmission of *banana bunchy top virus* to banana. Arch. Virol..

[12] Hogenhout SA, Ammar ED, Whitfield AE, Redinbaugh MG (2008). Insect vector interactions with persistently transmitted viruses. Annu. Rev. Phytopathol..

[13] Gray S, Cilia M, Ghanim M (2014). Circulative, “non propagative” virus transmission: An orchestra of virus-, insect-, and plant- derived instruments. Adv. Virus Res..

[14] Bernasconi ML, Turlings TCJ, Ambrosetti L, Bassetti P, Dorn S (1998). Herbivore-induced emissions of maize volatiles repel the corn leaf aphid, *Rhopalosiphum maidis*. Entomol. Exp. Appl..

[15] Pickett JA, Allemann RK, Birkett MA (2013). The semiochemistry of aphids. Nat. Prod. Rep..

[16] Piesik D, Pańka D, Jeske M, Wenda-Piesik A, Delaney KJ, Weaver DK (2013). Volatile induction of infected and neighbouring uninfected plants potentially influence attraction/repellence of a cereal herbivore. J. Appl. Entomol..

[17] Roosien BK, Gomulkiewicz R, Ingwell LL, Bosque-Pérez NA, Rajabaskar D, Eigenbrode SD (2013). Conditional vector preference aids the spread of plant pathogens: Results from a model. Environ. Entomol..

[18] Safari Murhububa I, Tougeron K, Bragard C, Fauconnier M-L, Basengere EB, Masamba JW, Hance T (2021). Banana tree infected with *Banana Bunchy Top virus* attracts *Pentalonia nigronervosa* aphids through increased volatile organic compounds emission. J. Chem. Ecol..

[19] McElhany P, Real LA, Power AG (1995). (1995) Vector preference and disease dynamics: A study of *Barley yellow dwarf virus*. Ecology.

[20] Ingwell LL, Eigenbrode SD, Bosque-Perez NA (2012). Plant viruses alter insect behavior to enhance their spread. Science.

[21] Rajabaskar D, Bosque-Pérez NA, Eigenbrode SD (2014). Preference by a virus vector for infected plants is reversed after virus acquisition. Virus Res..

[22] Carmo-Sousa M, Moreno A, Garzo E, Fereres A (2014). A non-persistently transmitted-virus induces a pull-push strategy in its aphid vector to optimize transmission and sprea. Virus Res..

[23] Carmo-Sousa M, Moreno A, Plaza M, Garzo E, Fereres A (2016). *Cucurbit aphid-borne yellows virus* (CABYV) modifies the alighting, settling and probing behaviour of its vector *Aphis gossypii* favouring its own spread. Ann. Appl. Biol..

[24] Eigenbrode SD, Bosque-Pérez NA, Davis TS (2018). Insect-borne plant pathogens and their vectors: Ecology, evolution, and complex interactions. Annu. Rev. Entomol..

[25] Koella JC, Sorensen FL, Anderson RA (1998). The malaria parasite, Plasmodium falciparum, increases the frequency of multiple feeding of its mosquito vector, *Anopheles gambiae*. Proc. R. Soc. Lond. Ser. B. Biol. Sci..

[26] Nault LR (1997). Arthropod transmission of plant viruses: A new synthesis. Ann. Entomol. Soc. Am..

[27] Ren G, Wang X, Chen D, Wang X, Fan X, Liu C (2015). Potato virus Y-infected tobacco affects the growth, reproduction, and feeding behavior of a vector aphid, *My. persicae* (Hemiptera: Aphididae). Appl. Entomol. Zool..

[28] Wohlers P, Tjallingii WF (1983). Electroantennogram responses of aphids to the alarm pheromone (E)-farnesene. Entomol. Exp. Appl..

[29] van Giessen WA, Fescemyer HW, Burrows PM, Peterson JK, Barnett OW (1994). Quantification of electroantennogram responses of the primary rhinaria of *Acyrthosiphon pisum* (Harris) to C4–C8 primary alcohols and aldehydes. J. Chem. Ecol..

[30] Nottingham SF, Hardy J, Dawson GW, Hick AJ, Pickett JA, Wadhams LJ, Bécasse CM (1991). Behavioral and electrophysiological responses of aphids to host and nonhost plant volatiles. J. Chem. Ecol..

[31] Hardie J, Visser JH, Piron PGM (1995). Peripheral odour perception by adult aphid forms with the same genotype but different host–plant preferences. J. Insect Physiol..

[32] Visser JH, Piron PGM (1998). Odour response profiles in aphids differentiating for species, clone, form and food. Proc. Exp. Appl. Entomol. Neth. Entomol. Soc. Amst..

[33] Park KC, Hardie J (1998). An improved aphid electroantennogram. J. Insect Physiol..

[34] Hazell SP, Gwynn DM, Ceccarelli S, Fellowes MDE (2005). Competition and dispersal in the pea aphid: Clonal variation and correlations across traits. Ecol. Entomol..

[35] Braendle C, Davis GK, Brisson JA, Stern DL (2006). Wing dimorphism in aphids. Heredity.

[36] Simmonds NW (1962). The Evolution of the Bananas. Tropical Science Series.

[37] Lorenzen J, Tenkouano A, Bandyopadhyay R, Vroh B, Coyne D, Tripathi L (2010). Overview of banana and plantain (*Musa* spp.) Improvement in Africa: Past and future. Acta Hortic..

[38] Ploetz RC (2015). Management of Fusarium wilt of banana: A review with special reference to tropical race 4. Crop. Prot..

[39] Williams IS, Dixon AFG, van Emden HF, Harrington R (2007). Life cycles and polymorphism. Aphid as Crop Pests.

[40] Dowiya NB, Rweyemamu CI, Maerere AP (2009). Banana (*Musa* spp. Colla) cropping systems, production constraints and cultivar preferences in eastern Democratic Republic of Congo. J. Anim. Plant Sci..

[41] Thomas, J. E., Iskra-Caruana, M. L. & Jones, D. R. Maladies des *Musa*-Le bunchy top du Bananier. Fiche technique N° 4. INIBA, Montpellier, France, 2 (1994).

[42] Lepoivre, P. Phytopathologie. Ed. De Broek. 427 (2003).

[43] Kwa M (2003). Activation de bourgeons latents et utilisation de fragments de tige du bananier pour la propagation en masse de plants en conditions horticoles in vivo. Fruits.

[44] Kwa, M. La culture et la multiplication des plants de bananier (*Musa* sp.), Connaissances et techniques de base. CARBAP, RD Congo. 13 (2009).

[45] Meutchieye, F. Fiche Technique de multiplication des bananiers par la méthode de PIF. SECAAR, Lomé, Togo, 15 (2009).

[46] Sadom L, Tomekpé K, Folliot M, Côte F-X (2010). Comparaison de l'efficacité de deux méthodes de multiplication rapide de plants de bananier à partir de l'étude des caractéristiques agronomiques d'un hybride de bananier plantain (*Musa* spp.). Fruits.

[47] Mbunzu BBN, Ngbengbu N, Ngongo MK (2019). Evaluation du potentiel de prolifération d’explants de différentes dimensions de bananier plantain (*Musa* sp. Cv. AAB) par la macropropagation en conditions semicontrôlées. Rev. Afr. Environ. Agric..

[48] van Regenmortel, M. H. V. *et al*. Virus taxonomy: Classification and nomenclature of viruses. In *Seventh report of the International Committee on Taxonomy of Viruses, San Diego, San Francisco, New York, Boston, London, Sydney and Tokyo* (Academic Press, 2000).

[49] Thomas, J. E., & Iskracaruana, M. L. Bunchy top. In *Deseases of, Abaca and Enset* (Jones, D. R., ed), 241–253 (CABI Press, 1999).

[50] Park KC, Hardie J (2002). Functional specialisation and polyphenism in aphid olfactory sensilla. J. Insect Physiol..

[51] Murdie G (1969). Some causes of size variation in the pea aphid, *Acyrthosiphon pisum* Harris. Ecol. Entomol..

[52] Lenth R, Singmann H, Love J, Buerkner P, Herve M (2018). Emmeans: Estimated marginal means, aka least-squares means. R Package Version.

[53] R Core, T (2022). R: A Language and Environment for Statistical Computing.

[54] Mayer RT, Inbar M, McKenzie C, Shatters R, Borowicz V, Albrecht U, Powell CA, Doostdar H (2002). Multitrophic interactions of the silverleaf whitefly, host plants, competing herbivores, and phytopathogens. Arch. Insect Biochem. Physiol..

[55] Medina-Ortega KJ, Bosque-Pérez NA, Ngumbi E, Jiménez-Martínez ES, Eigenbrode SD (2009). *Rhopalosiphum padi* (*Hemiptera*: *Aphididae*) responses to volatile cues from barley yellow dwarf virus-infected wheat. Environ. Entomol..

[56] Mauck KE, Bosque-Pérez NA, Eigenbrode SD, De Moraes C, Mescher MC (2012). Transmission mechanisms shape pathogen effects on host-vector interactions: Evidence from plant viruses (ed C Fox). Funct. Ecol..

[57] Mauck KE, Chesnais Q (2020). A synthesis of virus-vector associations reveals important defciencies in studies on host and vector manipulation by plant viruses. Virus Res..

[58] Eigenbrode SD, Gomulkiewicz R (2022). Manipulation of vector host preference by pathogens: Implications for virus spread and disease management. J. Econ. Entomol..

[59] Mauck KE, De Moraes CM, Mescher MC (2010). Deceptive chemical signals induced by a plant virus attract insect vectors to inferior hosts. PNAS.

[60] Tungadi T, Groen SC, Murphy AM, Pate AE, Iqbal J, Toby J, Bruce A, Cunniffe NJ, Carr JP (2017). *Cucumber mosaic virus* and its 2b protein alter emission of host volatile organic compounds but not aphid vector settling in tobacco. Virol. J..

[61] Harris FK (1977). An ingestion-egestion hypothesis of non-circulative virus transmission. Aphids As Virus Vectors.

[62] Hurd H (2003). Manipulation of medically important insect vectors by their parasites. Annu. Rev. Entomol..

[63] Lefèvre T, Thomas F (2008). Behind the scene, something else is pulling the strings: Emphasizing parasitic manipulation in vector-borne diseases. Infect. Genet. Evol..

[64] Bressan A, Watanabe S (2011). Immunofluorescence localisation of *Banana bunchy top virus* (family *Nanoviridae*) within the aphid vector, *Pentalonia nigronervosa*, suggests a virus tropism distinct from aphid-transmitted luteoviruses. Virus Res..

[65] Sicard A, Zeddamb J-L, Yvona M, Michalakisc Y, Gutiérreza S, Blanca S (2015). Circulative nonpropagative aphid transmission of nanoviruses: An oversimplified view. J. Virol..

[66] Moreno-Delafuente A, Garzo E, Moreno A, Fereres A (2013). Aplant virus manipulates the behavior of its whitefly vector to enhanceits transmission efficiency and spread. PLoS One.

[67] Patton M, Bak A, Sayre J, Heck M, Casteel C (2020). A polerovirus, *Potato leafroll virus*, alters plant–vector interactions using three viral proteins. Plant Cell Environ..

[68] Safari Murhububa I, Tougeron K, Bragard C, Fauconnier M-L, Mugisho Bugeme D, Bisimwa Basengere E, Walangululu Masamba J, Hance T (2023). The aphid *Pentalonia nigronervosa* (*Hemiptera*: *Aphididae*) takes advantage from the quality change in banana plant associated with *Banana bunchy top virus infection*. J. Econ. Entomol..

[69] Zhang Y, Wu K, Wyckhuys KAC, Heimpel GE (2009). Trade-offs between flight and fecundity in the soybean aphid (*Hemiptera*: *Aphididae*). J. Econ. Entomol..

[70] Khuhro NH, Biondi A, Desneux N, Zhang L, Zhang Y, Chen H (2014). Trade-off between flight activity and life-history components in *Chrysoperla sinica*. BioControl.

