# Supplementary material for: Preference of Pentalonia nigronervosa for infected banana plants tends to reverse after Banana bunchy top virus acquisition
Source: Sci Rep. 2024 Feb 5;14:2993. doi: 10.1038/s41598-024-53205-x (PMC10844331; doi:10.1038/s41598-024-53205-x)
Supplement: Supplementary file 1 — Supplementary Information. [file 41598_2024_53205_MOESM1_ESM.docx]

## **Preference of *Pentalonia nigronervosa* for infected banana plants tends to reverse after *Banana bunchy top virus* acquisition.**

**Ignace SAFARI MURHUBUBA^1, 3,4,6*^, Claude BRAGARD^2^, Kévin TOUGERON^5^, Thierry HANCE^1^**

**Supplementary Material**

**Table S1**: Raw data for the total number of secondary rhinaria (RN), Wing area (mm^2^), and Tibia Length (mm) of the viruliferous and non-viruliferous winged aphids used used in the tests of this work. HDB: healthy dessert banana; IDB: infected dessert banana; HPB: healthy plantain banana; IPB: infected plantain banana.

| **Repetition** | **Total number of secondary rhinaria** | | | | **Wing area (mm^2^)** | | | | **Tibia Length (mm)** | | | |
| --- | --- | --- | --- | --- | --- | --- | --- | --- | --- | --- | --- | --- |
|  | **HDB** | **IDB** | **HPB** | **IPB** | **HDB** | **IDB** | **HPB** | **IPB** | **HDB** | **IDB** | **HPB** | **IPB** |
| 1 | 16 | 14 | 23 | 14 | 1.1 | 0.74 | 1.03 | 1.05 | 1.06 | 0.84 | 1.02 | 0.93 |
| 2 | 20 | 19 | 19 | 18 | 1.11 | 0.72 | 1 | 0.78 | 1.13 | 0.9 | 1.06 | 0.79 |
| 3 | 17 | 17 | 22 | 14 | 1.05 | 0.78 | 0.89 | 1.89 | 1.12 | 0.86 | 0.94 | 0.92 |
| 4 | 17 | 16 | 25 | 15 | 1.08 | 0.88 | 0.88 | 0.72 | 1.12 | 0.94 | 0.91 | 0.8 |
| 5 | 22 | 20 | 18 | 21 | 1.08 | 0.9 | 0.9 | 0.9 | 1.1 | 0.87 | 0.95 | 0.91 |
| 6 | 20 | 17 | 19 | 18 | 1.08 | 0.89 | 0.92 | 0.8 | 1.08 | 0.96 | 0.98 | 0.8 |
| 7 | 21 | 19 | 19 | 20 | 1.14 | 0.9 | 0.93 | 0.88 | 1.24 | 1 | 0.98 | 0.85 |
| 8 | 10 | 15 | 18 | 18 | 0.88 | 0.7 | 0.81 | 0.67 | 0.9 | 0.81 | 0.93 | 0.7 |
| 9 | 18 | 17 | 19 | 20 | 1.15 | 0.87 | 1.24 | 0.68 | 1.29 | 0.93 | 1.2 | 0.75 |
| 10 | 18 | 18 | 21 | 20 | 0.9 | 0.77 | 0.78 | 0.65 | 1.03 | 0.86 | 0.81 | 0.83 |
| 11 | 16 | 14 | 17 | 16 | 0.9 | 0.9 | 0.76 | 0.84 | 0.99 | 0.99 | 0.87 | 0.82 |
| 12 | 17 | 15 | 20 | 18 | 0.89 | 0.76 | 1.1 | 0.87 | 1 | 0.84 | 1.15 | 0.88 |
| 13 | 19 | 17 | 22 | 21 | 1.1 | 0.8 | 1.35 | 0.68 | 1.1 | 0.8 | 1.22 | 0.72 |
| 14 | 19 | 18 | 20 | 21 | 0.97 | 0.82 | 0.77 | 0.77 | 1.03 | 0.91 | 0.84 | 0.82 |
| 15 | 17 | 16 | 17 | 20 | 1.03 | 0.7 | 1.1 | 1 | 1.1 | 0.82 | 1.15 | 0.92 |
| 16 | 16 | 16 | 20 | 20 | 1.15 | 0.8 | 1.04 | 0.7 | 1.29 | 0.87 | 1.12 | 0.82 |
| 17 | 14 | 14 | 22 | 19 | 0.95 | 0.81 | 0.81 | 0.87 | 1 | 0.84 | 0.89 | 0.82 |
| 18 | 18 | 16 | 23 | 18 | 1.11 | 0.79 | 0.99 | 0.71 | 1.18 | 0.86 | 0.99 | 0.79 |
| 19 | 16 | 14 | 23 | 18 | 1 | 0.8 | 1.12 | 1.11 | 1 | 0.88 | 1.19 | 1 |
| 20 | 13 | 15 | 20 | 19 | 1.05 | 0.83 | 1.05 | 0.83 | 1.02 | 0.92 | 1.14 | 0.78 |

**Table S2**: Mean number (± SE) of aphids (alate) attracted to a banana plant or a control, as well as associated statistical results (Student's t-tests and p-value). HDB - S: Attractiveness of aphids between Healthy Dessert Banana and potting soil only; HPB - S: between Healthy Plantain Banana and potting soil only; S - E: between potting soil only and empty control. Significant results (p <0.05) are in bold.

|  | **HDB - S** | | | |  | **HPB - S** | | | |
| --- | --- | --- | --- | --- | --- | --- | --- | --- | --- |
|  | **HDB** | **S** | **t** | **p** |  | **HPB** | **S** | **t** | **p** |
| Non-viruliferous | 6.80 ± 0.52 | 0 ± 0 | 5.6 | **<0.001** |  | 8.85 ± 0.57 | 0 ± 0 | 5.8 | **<0.001** |
| Viruliferous | 4.65 ± 1.06 | 0 ± 0 | 128.93 | **<0.001** |  | 4.6 ± 0.82 | 0 ± 0 | 127.94 | **<0.001** |


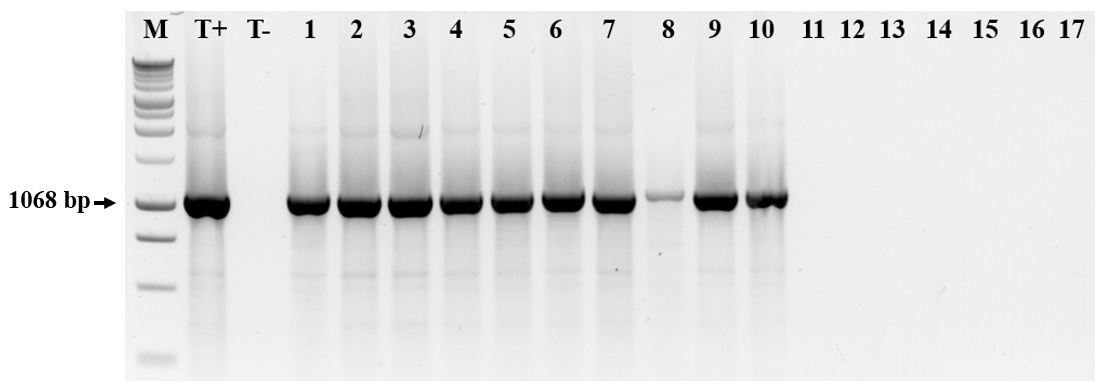


**Figure S1:** The viriliferous and non-viriliferous status, by PCR, of *P. nigronervosa* used in the tests. This test was carried out using the specific primers of *Banana bunchy top virus*: DNA-R-2drc Forward and DNA-R-2rdc Reverse, designed to amplify products of 1068 bp (F / TGCATTAGATGGTCTGGGAAA; R / ACTTCACGATGCCATGTTT).


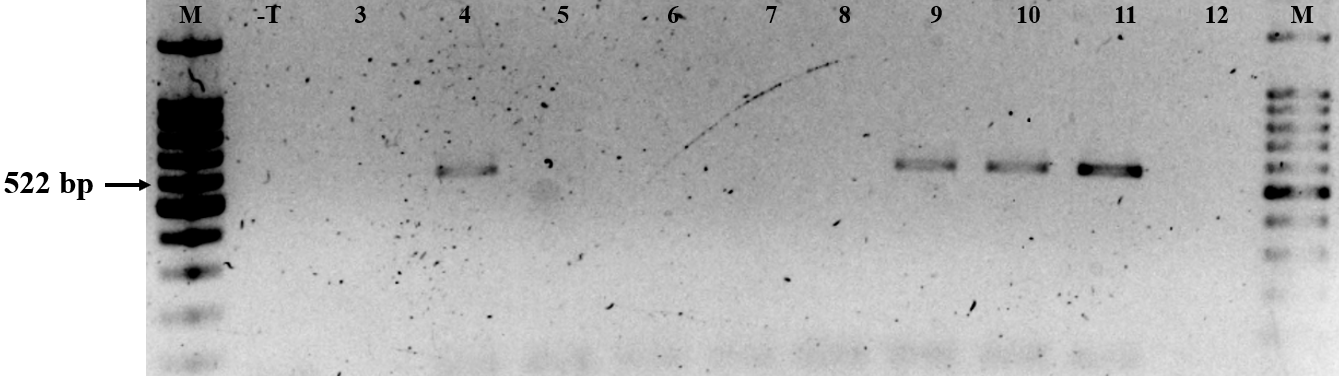


**Figure S2:** Discrimination (Genotyping) by PCR between dessert bananas and plantains. The control is a *Musa acuminata* banana with a known genotype: the Gros Michel variety (AAA). This test was carried out using the pair of Musa-OLF / Musa-OLR primers designated to target the junction between the banana genome and the eBSV (expected band at around 522 bp) (F / TGCATTAGATGGTCTGGGAAA; R / ACTTCACGATGCCATGTTT).


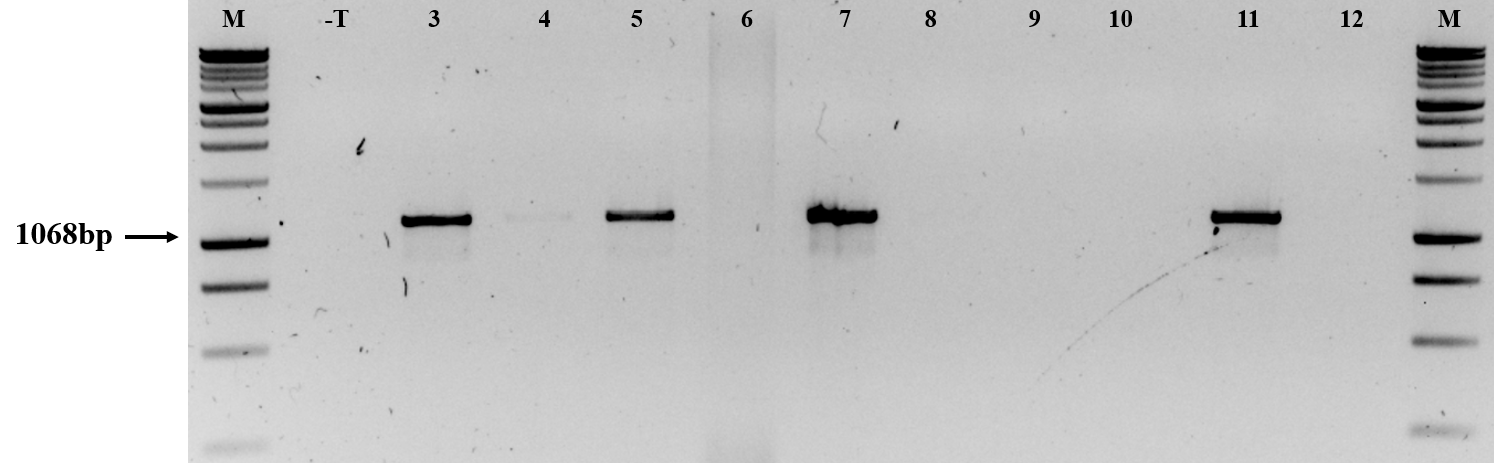


**Figure S3:** The health status, by PCR, of the banana strains used in the tests. The control is a well-known affected banana: the infected Gros Michel variety (AAA). This test was carried out using the specific primers of *Banana bunchy top virus*: DNA-R-2drc Forward and DNA-R-2rdc Reverse, designed to amplify products of 1068 bp (F / TGCATTAGATGGTCTGGGAAA; R / ACTTCACGATGCCATGTTT).
